# Influence of CAD/CAM Milling and 3D-Printing Fabrication Methods on the Mechanical Properties of 3-Unit Interim Fixed Dental Prosthesis after Thermo-Mechanical Aging Process

**DOI:** 10.3390/polym14194103

**Published:** 2022-09-30

**Authors:** Passent Ellakany, Shaimaa M. Fouda, Amr A. Mahrous, Maram A. AlGhamdi, Nourhan M. Aly

**Affiliations:** 1 Department of Substitutive Dental Sciences, College of Dentistry, Imam Abdulrahman Bin Faisal University, Dammam 32210, Saudi Arabia; 2 Department of Pediatric Dentistry and Dental Public Health, Faculty of Dentistry, Alexandria University, Alexandria 21527, Egypt

**Keywords:** mechanical properties, CAD/CAM, milling, 3D-printing, PMMA, interim IFDP

## Abstract

This study assessed the influence of CAD/CAM milling and 3D-printing fabrication methods on mechanical properties of 3-unit interim fixed dental prosthesis (IFDPs) after thermo-mechanical aging. Forty 3-unit IFDPs were fabricated on a mandibular right second premolar and second molar of a typodont cast. Samples were fabricated from the following materials; auto-polymerized polymethyl methacrylate (conventional resin), CAD/CAM PMMA (milled resin) and two different CAD/CAM 3D-printed composite resins; digital light processing Asiga (DLP AS) and stereolithography NextDent (SLA ND). Mechanical properties were compared between the studied materials using Kruskal–Wallis test, followed by multiple pairwise comparisons using Bonferroni adjusted significance. There was a significant difference in flexural strength and microhardness between the studied materials (*p* < 0.001), with the highest mean ± SD reported in the milled IFDPs (174.42 ± 3.39, 27.13 ± 0.52), and the lowest in the conventional IFDPs (98.02 ± 6.1, 15.77 ± 0.32). Flexural strengths differed significantly between the conventional IFDPs and all materials except DLP AS. The highest elastic modulus was recorded in the milled group, and the lowest in the SLA ND group (*p* = 0.02). In conclusion, superior flexural strength, elastic modulus, and hardness were reported for milled IFDPs. SLA ND printed IFDPs showed comparable mechanical properties to milled ones except for the elastic modulus.

## 1. Introduction

Interim fixed dental prosthesis (IFDP) is subjected to intraoral thermal changes and occlusal loads that, in the long-term, might cause its distortion [1,2]. Moreover, comprehensive treatment plans that include oral rehabilitation, extensive occlusal reconstruction or implant supported prosthesis might require the use of IFDP for a longer time [3,4,5]. Thus, mechanical properties of IFDP are of paramount importance in such situations [6,7,8] as they affect the longevity of the material used for construction, particularly in restoring long edentulous spans [7,9].

Auto-polymerized polymethyl methacrylate (PMMA) is the most common material used in construction of IFDPs [10,11]. It is a readily available, inexpensive, and easy to use material [12,13]. Nevertheless, it requires laboratory and chairside adjustments [6] in addition to the manual mixing of the powder and monomer that results in stress concentrations and entrapment of air bubbles that make PMMA more prone to water sorption and mechanical failure [12,13,14,15].

Computer-aided design and computer-aided manufacturing (CAD/CAM) technology is increasingly used in dentistry [13]. Nowadays, this technology is more available, and widely used in the fabrication of IFDPs [16,17]. Manufacturing of dental prostheses using CAD/CAM technology could be accomplished by subtractive method (milling), or additive method (three-dimensional (3D) printing) [8,18,19]. Milled resin is currently used in the fabrication of IFDPs because it has shown higher strength and durability than self-cured PMMA before and after thermomechanical aging [6,12,20]. However, the high expense of this technology might limit its use [21,22].

Additive 3D-printing technology is another method used for fabrication of IFDPs using CAD/CAM technology at a lower price [23] with less material waste and fabrication time [24,25,26]. Additionally, 3D-printing could improve patient acceptance, provide adequate marginal and internal fit at reduced expenses [27,28]. Several materials are being used by 3D-printing technology such as dental ceramics, composites, polymer resins as polyether etherketone (PEEK) and PMMA and metals as titanium, stainless steel and Cr-Co alloys [29]. All these materials allowed the extensive use of 3D-printing technology these days in the fabrication of surgical guided stents, diagnostic casts, custom trays, wax patterns and dental implants. Moreover, complete dentures can be fabricated by the printing technology for a short term use [30]. However, 3D-printing is still suffering from certain disadvantages as polymerization, shrinkage, high cost of resin and machines in comparison to conventional methods of fabrication, and surface roughness from layering deposition of resin with the production of toxic resin wastes [18,31].

The most widely used 3D-printing technologies in the fabrication of 3D-printed dental prostheses are stereolithography (SLA) and digital light projection (DLP). The main differences between both 3D-printing technologies are the materials used and technique of building layers to create a 3D-object. SLA is preferred over DLP because of its higher accuracy in producing detailed objects, and smooth surface [32]. However, SLA has several drawbacks such as limited longevity and low fracture strength. Moreover, DLP is faster than SLA in the fabrication of printed objects and wastes less material; thus reducing the cost [33].

Additive manufacturing technologies have showed significant improvement in the fabrication procedures and associated materials. Development of polymeric ceramic composite (PCC) has been used successfully in fabrication of 3D-printed prosthetic restorations of high esthetics and overcoming the disadvantages of dental ceramics such as abrasion of opposing natural teeth. However, adhesion of polymer resin, ceramics and fillers in the matrix is still challenging, where failure of this adhesion can lead to the production of rough surfaces and reduced mechanical properties of dental prostheses. These problems were overcome by the addition of alumina and zirconia ceramic fillers to improve the adhesion process [34]. A previous article used the same fillers with application of etchant, a silane coupling agent and the calcination process at various temperatures to enhance the adhesion mechanism [35]. However, the addition of the previously mentioned fillers needs further studies to determine the acceptable amount of filler and degree of thermal treatment that can be added to PCC without compromising the mechanical properties of the prosthetic restorations. In another study, 3D-printed temporary resins were reinforced by the addition of zirconium oxide (ZrO_2_) nanofillers, which showed a significant increase of flexural strength and hardness of 3D-printed resins in comparison to unfilled resins [30]. Additionally, it resulted in the production of an esthetic biocompatible restorative material matching the translucency of natural dentition and exhibiting antibacterial and antifungal properties [36,37].

Information on the mechanical properties and clinical behavior of IFDPs fabricated by milling and 3D-printing technologies is under controversy [8,9,38,39,40]. Meanwhile, few studies assessed the impact of thermomechanical aging on the elastic modulus of milled and 3D-printed IFDPs [23,38]. Therefore, the aim of the current study was to assess the mechanical properties of CAD/CAM milled and two different types of 3D-printed, 3-unit IFDPs in comparison to the conventional IFDPs after the thermo-mechanical aging process. The first hypothesis stated that milled and 3D-printed IFDPs are of comparable mechanical properties to conventional IFDPs. The second hypothesis stated that SLA and DLP 3D-printing technologies would have similar impacts on the mechanical properties of 3D-printed IFDPs after the thermomechanical aging process.

## 2. Materials and Methods

### 2.1. Master Model Preparation

**Figure 1:**
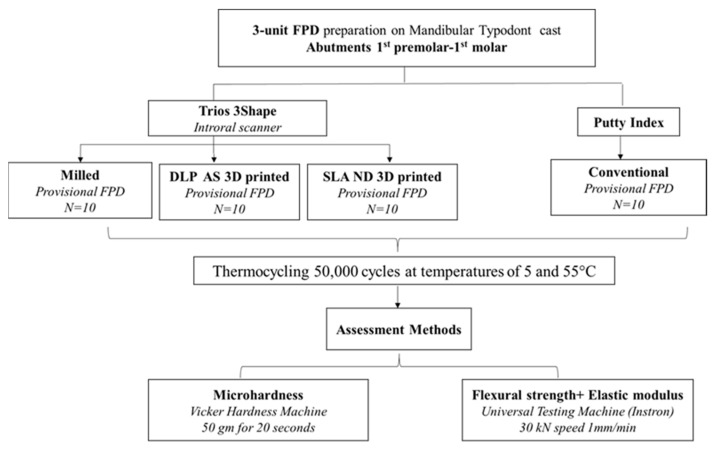
Flow chart of the study design.

Four different interim dental restorative materials were used in the current study to fabricate 3-unit IFDP on a mandibular right second premolar and second molar. The abutments were prepared on a mandibular typodont cast (D85DP-500B.1, Nissin Dental Product Inc., Kyoto, Japan). The samples of 3-unit IFDP were fabricated from the following materials: auto-polymerized PMMA (conventional resin), CAD/CAM PMMA (milled resin) and two different CAD/CAM 3D-printed composite resins; DLP AS and SLA ND; as shown in Table 1 and Figure 1. The required sample size was calculated assuming 80% study power and 5% alpha error. Digholkar et al. [8] reported mean ± SD flexural strength of 3D-printed, milled, and conventional resins = 95.88 ± 12.44, 79.54 ± 10.13, and 104.20 ± 12.78, respectively. Based on comparison of means, the minimum sample size was calculated to be 9 per group, increased to 10 to make up for laboratory processing errors (G*Power v3.1.9.2; Heinrich Heine University Düsseldorf) [41,42]. Ten IFDPs (n = 10 × 4) were fabricated from each material studied according to the manufacturers’ instructions. Thus, the total required sample size was 40 IFDPs. All samples were of A2 shade.

A mandibular first molar typodont tooth (pontic) was placed on the typodont cast to make polyvinyl siloxane molds (Express STD; 3M ESPE, St Pau, MN, USA) for the fabrication of conventional 3-unit IFDP samples on the mandibular second premolar, first molar and second molar before teeth preparation. The mandibular first molar was then removed and blocked with baseplate wax to create the socket region that will be substituted with the pontic of the 3-unit IFDP. Abutments’ preparation was done using a round-ended diamond stone in the following dimensions: occlusal reduction of 2 mm, axial reduction 1.5 mm in thickness with 6 degree occlusal convergence and the chamfer finish line was 1 mm thick all around and positioned 1 mm occlusal to the gingival margin and rounded internal line angles [9].

### 2.2. Fabrication of the Conventional 3-Unit IFDPs

Following the abutments’ preparation, a layer of petroleum jelly was applied on the prepared surface of the abutments and the typodont cast to facilitate removal of the IFDPs. Fabrication of conventional IFDPs was achieved through manual mixing of powder and monomer on a glass slab with a mixture ratio 1:1. Conventional resin mix was then loaded on the silicone mold to be seated on the prepared teeth. Complete setting of the IFDPs was reached after at least 5 min at room temperature [25]. Finishing and polishing of the IFDPs were done using silicon carbide abrasive discs of decreasing grits 360, 600 and 800 under a water cooling system [13].

### 2.3. Fabrication of Milled 3-Unit IFDP

Fabrication of milled IFDPs was initiated by scanning the typodont casts by an intraoral scanner (Trios 3Shape Dental Software, 3Shape A/S, Copenhagen, Denmark) to create a standard tessellation language (STL) file. Designing of 3-unit IFDP was done using CAM designing software (3Shape Dental Software, 3Shape A/S, Copenhagen, Denmark). Distance between the two retainers was set to be 20 mm as in Zimmermann et al.’s [43] study. The dimensions of the connectors between abutment teeth were designed to be 4 × 4 mm, and the pontic height was 9 mm [44]. The designed STL file was sent to the integrated milling machine (PM7, Ivoclar, Vivadent, Schaan, Liechtenstein) to fabricate 10 IFDPs from millable blocks (Table 1). Minor adjustments were done through removal of samples from the supporting struts.

### 2.4. Fabrication of 3-Printed 3-Unit IFDP by 2 Printing Technologies

**Figure 2:**
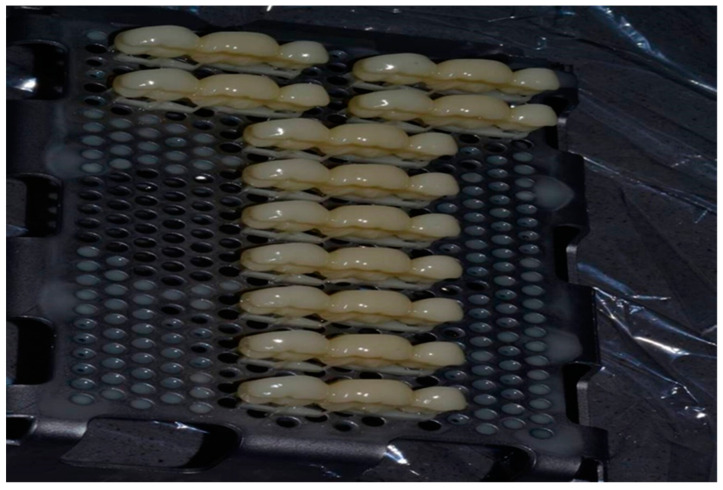
Printing orientation of IFDP (90°).

The same designed STL file mentioned before was sent to two different 3D-printers; Nextdent printer (based on SLA technology) and ASIGA printer (based on DLP technology). Both printers fabricated the same dimensions of 3-unit IFDPs from two different 3D-printed composite resins (SLA ND and DLP AS resins) at 90-degree building orientation and 50 µm layer thickness (Figure 2).

Then, the IFDPs were immersed in 99% isopropyl alcohol for 1 min followed by application of compressed air to achieve dry spraying. The same procedure of alcohol immersion was repeated, but for 50 s only [39]. A post-curing phase was done according to the manufacturer’s instructions of each technology as mentioned in (Table 1). Then, polishing of specimens was done as mentioned in the fabrication of conventional IFDPs. The dimensions of all fabricated specimens from the four different materials were evaluated by a digital caliper (Digital ABS AOS Caliper; Mitutoyo Corp, Tokyo, Japan) according to the designed dimensions. According to previous articles, the IFDPs were not fixed with dental cements to avoid any additional variables that might affect the findings, since the use of dental cements allows even distribution of the applied force on IFDPs and increases their resistance to fracture [12,45,46,47]. The IFDPs were placed in distilled water at a temperature of 37 °C for 24 h [39].

### 2.5. Thermomechanical Aging of All Study Materials of 3-Unit IFDP

All IFDPs underwent thermomechanical aging for 50,000 cycles between chambers at temperatures of 5 and 55 °C, with 60 s dwell time and five seconds transfer time using a thermocycling machine (Thermocycler THE-1100-SD Mechatronik GmbH, Feldkirchen-Westerham, Germany), to simulate 6 months intraoral conditions [21].

### 2.6. Flexural Strength and Elastic Modulus of 3-Unit IFDP

**Figure 3:**
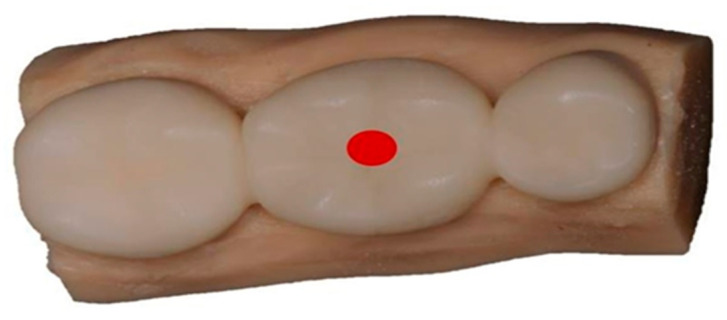
Location of metal jig of instron machine on the 3-unit IFDP sample (red circle on the central fossa of oclusal surface).

In order to assess the 3-point bend flexural strength and elastic modulus of the 3-unit IFDPs, a metal jig was directed towards the center of the occlusal surface of the pontic parallel to the long axis of the tooth [44] using a calibrated universal testing machine (Instron 8871; Instron Co., Norwood, MA, USA) (Figure 3). An axial load of 30 kN was applied with a crosshead speed of 1 mm/min until failure was reached [9]. Fracture force was recorded once samples were broken. Calculation of the flexural strength and elastic modulus were done in (MPa) in accordance with the International Organization for Standardization (ISO10477-2018 standard) [48] through the following equations:

Flexural strength = 3FL/2bh^2^

where (F) represents the fracture force in Newton (N), (L) represents the distance between the 2 supporting arms, (b) represents the width of the tested specimen and (h) represents the thickness.

Elastic modulus = FL^3^/4bh^3^d

where (F) represents the load applied on the linear segment of the stress strain curve in (N), and (d) is the deflection recorded at (F) [13].

### 2.7. Surface Microhardness Test

Surface microhardness of the fractured IFDPs was assessed by the hardness tester machine (MicroMet 6040, Buehler LTD., Lake Bluff, IL. USA) through application of a 50 g loading force for 20 s (Standard-ASTM C1327–03) using the diamond Vickers hardness indenter (VH) [49]. An average of five indentations on different areas of each specimen was calculated. The indentation readings were detected by a single trained operator (P.E). Calculation of VH was done through the following equation:

KHN = 14,228c/d^2^

where (KHN) represents the Knoop hardness readings, (c) represents the applied force in grams and (d) represents the length of the longest diagonal detected in mm [40].

### 2.8. Statistical Analysis

Means and standard deviations (SD) were calculated for all variables. Normality was tested for all variables using descriptive statistics, plots, and normality tests. All variables showed non-normal distribution, so non-parametric tests were used. Comparisons between the 4 studied materials were done using Kruskal-Wallis test, followed by multiple pairwise comparisons (in case of significant results) using Bonferroni adjusted significance level. Significance was inferred at *p* = 0.05. Data were analyzed using IBM SPSS for Windows (Version 23.0).

## 3. Results

**Figure 4:**
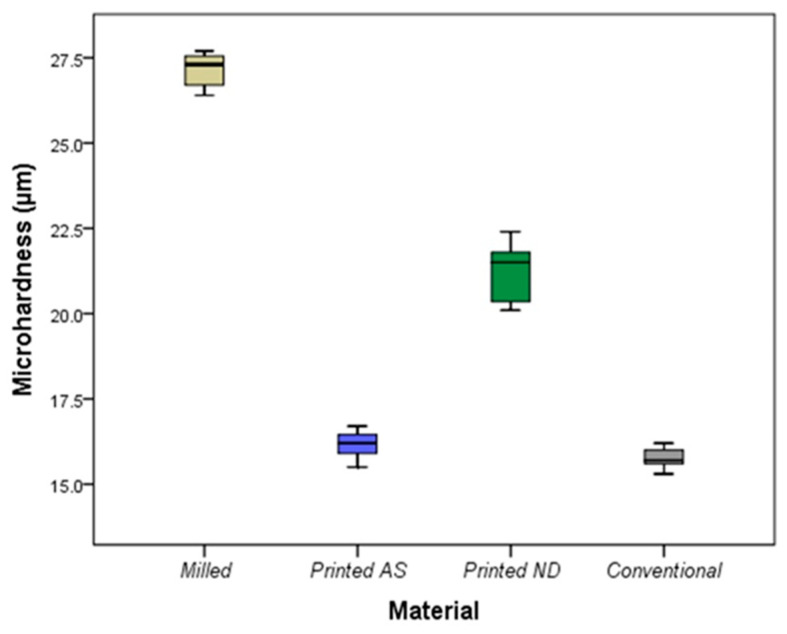
Microhardness of the studied materials. Middle line represents the median, box outline represents the interquartile range (25th–75th percentiles), and the two whiskers represent the range (minimum–maximum).

Table 2 and Table 3 show the comparisons of mechanical properties between the studied materials. There was a significant difference in microhardness between the studied materials (*p* < 0.001) with the highest mean ± SD reported in the milled IFDPs (27.13 ± 0.52), and the lowest reported in the conventional IFDPs (15.77 ± 0.32) (Figure 4). Post hoc comparisons showed a significant difference between milled and DLP AS (*p* = 0.002), and no difference between DLP AS and SLA ND (*p* = 0.30). The only material that did not differ significantly from the conventional group was the DLP AS (*p* = 1.00). Additionally, there was a significant difference in force at break between the tested materials (*p* < 0.001) with the highest mean ± SD reported in the milled IFDPs (1794.06 ± 34.83), and the lowest reported in the conventional IFDPs (1008.23 ± 62.87) (Table 2).

**Figure 5:**
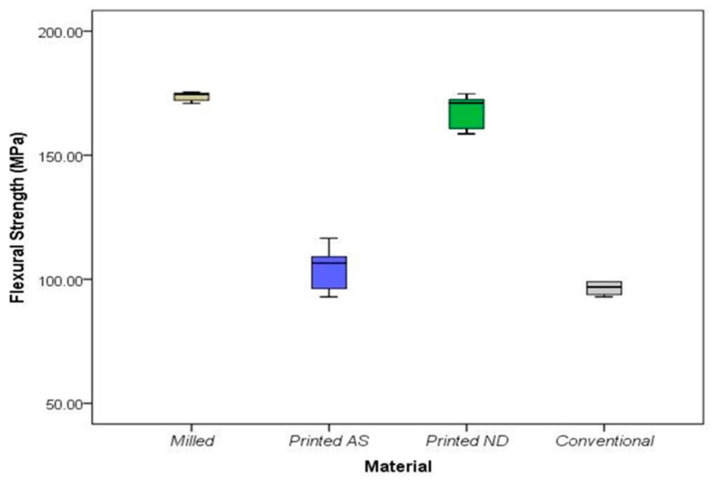
Flexural strength of the studied materials. Middle line represents the median, box outline represents the interquartile range (25th–75th percentiles), and the two whiskers represent the range (minimum–maximum).

Post hoc comparisons showed a significant difference between milled and DLP AS (*p* = 0.005), and no difference between DLP AS and SLA ND (*p* = 0.08) (Table 3). The only material that did not differ significantly from the conventional group was the DLP AS (*p* = 1.00). Moreover, there was a significant difference in flexural strength between the studied materials (*p* < 0.001). The highest mean flexural strength was recorded in the milled group (mean ± SD = 174.42 ± 3.39), while the lowest was recorded in the conventional group (mean ± SD = 98.02 ± 6.11) (Figure 5). Post hoc comparisons showed a significant difference between milled and DLP AS (*p* = 0.005), but no difference between DLP AS and SLA ND (*p* = 0.08). Additionally, the flexural strengths differed significantly between the conventional IFDPs and all studied materials except DLP AS.

**Figure 6:**
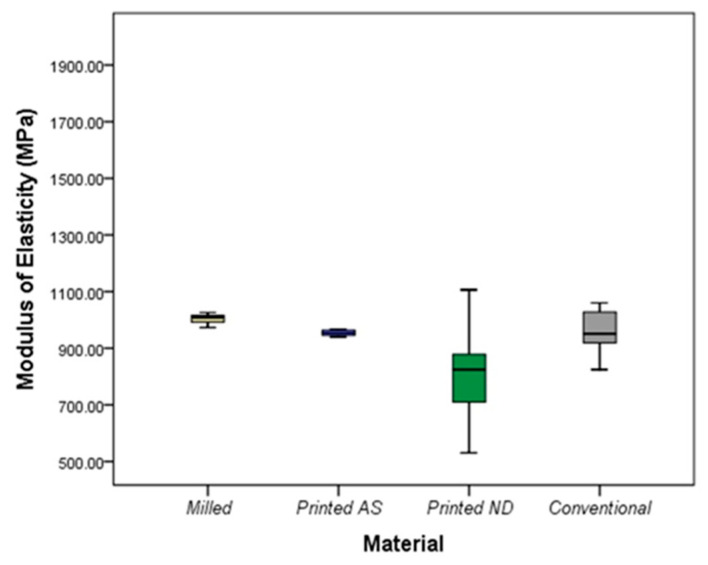
Elastic modulus (MPa) of the studied materials.

**Figure 7:**
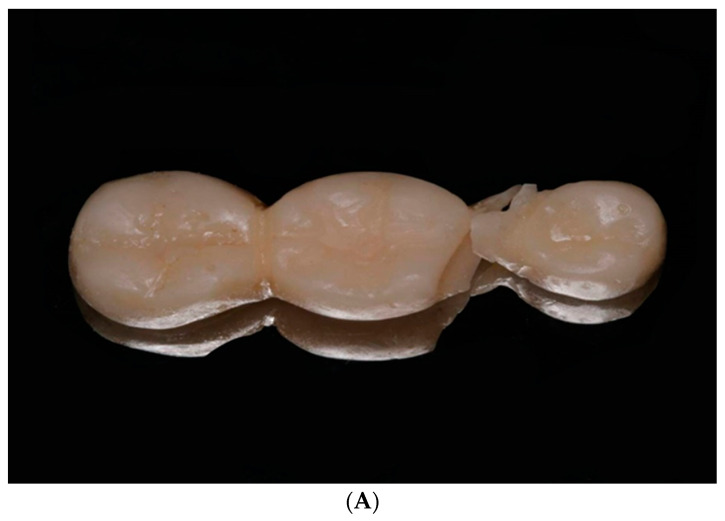

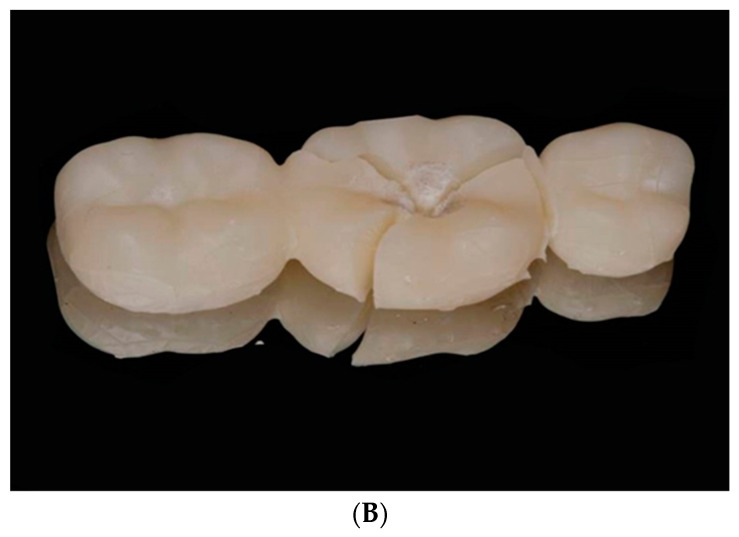
(**A**) Fracture pattern of IFDP on connectors among milled, DLP AS and conventional samples. (**B**) Fracture pattern of IFDP on pontic and connector regions among SLA ND samples.

Regarding the elastic modulus, the highest mean value was recorded in the milled group (mean ± SD = 1003.71 ± 18.51), while the lowest was reported in the SLA ND group (mean ± SD = 805.47 ± 190.37) with a significant difference between both groups (*p* = 0.02) with no significant differences between other groups (Figure 6). All IFDPs subjected to static load were fractured at the connector region (Figure 7A) except SLA ND specimens that exhibited catastrophic fractures at the pontic and connector regions (Figure 7B).

## 4. Discussion

The present study compared the mechanical properties of milled and 3D-printed IFDPs to the conventional samples after the thermomechanical aging process. The first study hypothesis was partly rejected because there were significant differences between the tested properties of milled and DLP AS printed IFDPs and the elastic modulus of milled and SLA ND samples, while the second hypothesis was accepted because 3D-printed IFDPs fabricated by SLA and DLP technologies showed comparable properties after thermomechanical aging process.

In agreement with previous studies, the flexural strength and force at fracture of milled IFDPs was higher than the conventional group [1,6,12,13,21]. The tested materials in the present study were subjected to thermal cycling. Previous studies showed that milled IFDPs were less susceptible to hydrolytic degradation and fracture supporting the present results [13,21,39]. This might be explained by the high load bearing capacity of the polymer structure of milled resins, which are fabricated under high pressure and temperature [17], so milled IFDPs would require more force to fracture, as reported in the current study. Additionally, it is less porous, free of voids and residual monomer, and has a highly cross-linked structure in contrast to the linear polymer structure, high polarity, and air bubbles entrapped in the conventional samples during manual mixing [13,14]. All these factors might reduce the water sorption and plasticizing effects among milled IFDPs as previously reported [5,9,13,14]. Furthermore, the strength of the conventional IFDPs was decreased due to the induced plasticizing effect on the polymer networks leading to degradation of the polymeric chains by hydrolysis of the monomer.

The flexural strength and force at break of milled resins in the present study were comparable to SLA ND, but significantly higher than DLP AS. Similarly, Henderson et al. [25] found higher flexural strength of the 3-unit milled IFDPs than 3D printed ones. Additionally, in agreement with these results, Tasin et al. [13] reported that the flexural strength of SLA ND did not differ significantly from the milled resins. The reason for the high strength of SLA ND could be the presence of microfillers in its composition in addition to the mode of fabrication, which was SLA printing technology. In SLA, the printing of successive layers is achieved through controlled penetration of an UV laser beam released from the focal spot, while in DLP, the whole printed layer is polymerized through reflection of the laser beam towards a mirror. Thus, more stepwise effects might result on DLP-printed objects, resulting in reduced mechanical properties when compared to SLA structures [23].

Conversely, Digholkar et al. [8] reported lower flexural strength of 3D-printed microhybrid-filled composite resins when compared to milled resins. This difference might be related to the use of different 3D-printing material and tested bar shape specimens unlike the 3D-printed 3-unit IFDP tested in the present study. On the contrary, Suralik et al. [39] reported higher flexural strength of 3D-printed specimens than that of milled ones. This variation shows that not only the material composition and testing procedures could affect the findings, but also the use of different printing orientations, printer, milling machines and the printing parameters recommended by the manufacturer for each 3D-printed resin [4,13,23].

In assessing the elastic modulus of the tested resins, the unfilled 3D-printed resin (DLP AS) used in this study showed comparable results to conventional and milled resins. The elastic modulus of SLA ND was significantly lower than milled, but comparable to conventional resins. In contrast, Taheyeri et al. [23] reported a significantly higher elastic modulus in filled conventional resins than unfilled 3D-printed and conventional resins, while both unfilled resins were of equivalent moduli that allow their use intraorally. This difference might be related to the difference in composition of the used materials or the testing protocol. All specimens were fractured mainly at the connector region except SLA ND specimens, where the fracture was extended to the pontic region. This variation between the studied materials might be related to the value of elastic modulus since the decrease in elastic modulus leads to reduced resistance to fracture [47]. This finding agreed with the results of previous studies that tested the flexural strength of 3- or 4-unit prostheses [11,12,14,19,50]. However, Henderson et al. [25] reported that the fracture of the IFDP was restricted to the distal connector extending to the pontic cusp tip. The difference in fracture pattern is related to the size of the connector and the direction of applied load on the external surface of the IFDP. The premolar and molar retainers of the IFDPs were not subjected to fracture. This explains the need to fabricate 3-unit IFDPs with a strong connector, pontic design and dimensions particularly in long spans IFDPs to reduce the possibility of fracture when subjected to functional and masticatory loads [47]. Accordingly, both 3D-printed resins (the unfilled DLP AS and the microfilled SLA ND) would have sufficient mechanical properties that are comparable to conventional resins allowing their use in fabrication of intraoral interim prostheses.

Milled IFDPs showed the highest hardness which did not differ significantly from SLA ND ones, but was significantly higher than DLP AS and conventional IFDPs, which were of comparable hardness. Similar to a previous report, milled IFDPs showed significantly higher surface hardness than conventional IFDPs [6]. On the contrary, other studies reported higher hardness for 3D-printed resins than conventional and milled resins [3,8]. They suggested that the high hardness of 3D-printed resins might result from the presence of cross-linked monomers and inorganic fillers, that increased its abrasion resistance [3,8,10]. This suggestion supports the non-significant difference in hardness between mircofilled SLA ND and milled IFDPs. However, the variation between the current findings and previous studies might be attributed to the difference of the tested CAD/CAM and conventional resins, difference in printing technology and design of the tested specimens. In addition, the present study included two types of 3D-printed resins with various chemical compositions and printing methods that showed different performance for the 3D-printed resins.

The IFDPs tested in this study were subjected to thermal cycling to mimic the intraoral temperature changes. Moreover, two types of 3D-printed resins fabricated by different processing technologies (SLA and DLP) were included in this study in addition to milled resins. However, the effect of occlusal loads was not simulated in this in vitro study. The materials were tested under static loads, and results may be different when dynamic loads are used. Although in vitro studies allow clinicians to compare the performance of materials under standardized conditions, long-term clinical studies are required for the better understanding of behavior of different interim materials.

## 5. Conclusions

Despite the limitations of the current study, it was concluded that superior flexural strength and hardness were reported in milled IFDPs compared to SLA ND, DLP AS and conventional IFDPs. This might be related to the reduced hydrolytic degradation susceptibility and fracture of milled resins resulting from the fabrication of blocks under high pressure and temperature. SLA ND-printed IFDPs showed comparable mechanical properties to milled ones, except for the elastic modulus, which showed a significantly lower value. The presence of microfillers in the composition of SLA ND and the mode of fabrication that depends on SLA printing technology might provide a valid explanation for the improved mechanical properties of SLA technology over DLP. DLP AS showed comparable properties to conventional IFDPs, but had significantly lower mechanical properties when compared to milled ones, except for the elastic modulus, which was similar in both groups. Fractures mainly occurred in the connector region for all materials except SLA ND, which also extended to the pontic region. This shows the high demand to fabricate 3-unit IFDPs with a strong connector, pontic design and dimensions, particularly in long spans IFDPs to withstand regular functional and masticatory loads and resist fracture. In conclusion, all tested materials are suitable for clinical application following a thermal mechanical aging process with greater privilege to the milled and SLA ND-printed IFDPs due to their significantly higher fracture resistance when compared to the other tested materials.

## Figures and Tables

**Table 1:** Materials used in the current study.

Interim Materials Used	Composition	Abbreviation	Trade Name and Manufacturer	Type of Machine Used	Manufacturing Technique	Laser Beam Intensity	Post Curing Phase
**Auto-polymerized polymethyl methacrylate resin (auto-polymerized PMMA)**	Methyl methacrylates N, N-diméthyl-p-toluidine Methyl methacrylate and ethyl methacrylate copolymer	Conventional resin	Unifast Trad, GC chemicals, Tokyo, Japan	-	Manual	-	-
**CAD/CAM PMMA blocks**	Cross-linked Polymethyl methacrylate-based polymer	Milled resin	Telio CAD; Ivoclar Vivadent	CAM milling machine (PM 7, Ivoclar, Vivade)	Milling	-	-
**3D-printed composite resins**	Microfilled Methacrylic oligomers & phosphine oxides	SLA ND resin	NextDent C&B MFH, Soesterburg, The Netherlands	405 nm UV LED NextDent 5100 3D	3D printing SLA	405 nm UV LED	LC-D Print Box, 3D systems, Vertex Dental B.V., Soesterberg, Netherland
**3D-printed composite resin**	Photopolymerized Methacrylate resin	DLP AS resin	ASIGA DentaTooth, ASIGA, Erfurt, Germany	Asiga MAX UV	3D printing DLP	UV LED (385–405 nm)	Asiga Flash UV Curing Chamber, Erfurt, Germany

**Table 2:** Comparison of the mechanical properties between the studied materials.

	Milled	DLP AS	SLA ND	Conventional	KWT *p* Value
	Mean ± SD				
**Microhardness (µm)**	27.13 ± 0.52	16.16 ± 0.43	21.19 ± 0.91	15.77 ± 0.32	<0.001 *
**Force at break (N)**	1794.06 ± 34.83	1067.57 ± 91.85	1720.26 ± 71.18	1008.23 ± 62.87	<0.001 *
**Flexural strength (MPa)**	174.42 ± 3.39	103.79 ± 8.93	167.25 ± 6.92	98.02 ± 6.11	<0.001 *
**Elastic modulus (MPa)**	1003.71 ± 18.57	951.13 ± 68.61	805.47 ± 190.37	961.48 ± 84.76	0.04 *

KWT: Kruskal–Wallis test was used. * Statistically significant at *p* value < 0.05.

**Table 3:** Post hoc comparisons between different studied materials and tests.

	Group	Compared to	*p* Value
**Microhardness**	Milled	DLP AS	0.002 *
		SLA ND	0.67
		Conventional	<0.001 *
	DLP AS	SLA ND	0.30
		Conventional	1.00
	SLA ND	Conventional	0.03 *
**Force at break**	Milled	DLP AS	0.005 *
		SLA ND	1.00
		Conventional	0.001 *
	DLP AS	SLA ND	0.08
		Conventional	1.00
	SLA ND	Conventional	0.02 *
**Flexural strength**	Milled	DLP AS	0.005 *
		SLA ND	1.00
		Conventional	0.001 *
	DLP AS	SLA ND	0.08
		Conventional	1.00
	SLA ND	Conventional	0.02 *
**Elastic modulus**	Milled	DLP AS	0.86
		SLA ND	0.02 *
		Conventional	1.00
	DLP AS	SLA ND	0.92
		Conventional	1.00
	SLA ND	Conventional	0.51

* Statistically significant at Bonferroni adjusted significance level.

## Data Availability

Data is available upon request from the corresponding author.

## References

[1] Akiba S., Takamizawa T., Tsujimoto A., Moritake N., Ishyii R., Barkmeier W., Latta M.A., Miyazaki M. (2019). Influence of different curing modes on flexural properties, fracture toughness, and wear behavior of dual-cure provisional resin-based composites. Dent. Mater. J..

[2] Pascutti F., Kreve S., Carvalho G., Grecco P., Franco A., Dias S. (2017). Evaluation in vitro of Flexural Strength of Three Resins for Provisional Crowns in CAD/CAM System. J. Dent. Sci..

[3] Al-Qahtani A.S., Tulbah H.I., Binhasan M., Abbasi M.S., Ahmed N., Shabib S., Farooq I., Aldahian N., Nisar S.S., Tanveer S.A. (2021). Surface Properties of Polymer Resins Fabricated with Subtractive and Additive Manufacturing Techniques. Polymers.

[4] Perea-Lowery L., Gibreel M., Vallittu P.K., Lassila L. (2020). Characterization of the mechanical properties of CAD/CAM polymers for interim fixed restorations. Dent. Mater. J..

[5] Liebermann A., Wimmer T., Schmidlin P.R., Scherer H., Löffler P., Roos M., Stawarczyk B. (2016). Physicomechanical characterization of polyetheretherketone and current esthetic dental CAD/CAM polymers after aging in different storage media. J. Prosthet. Dent..

[6] Rayyan M.M., Aboushelib M., Sayed N.M., Ibrahim A., Jimbo R. (2015). Comparison of interim restorations fabricated by CAD/CAM with those fabricated manually. J Prosthet. Dent..

[7] Shim J., Kim H., Park S., Yun H., Ryu J. (2019). Comparison of Various Implant Provisional Resin Materials for Cytotoxicity and Attachment to Human Gingival Fibroblasts. Int. J. Oral. Maxillofac. Implants.

[8] Digholkar S., Madhav V.N.V., Palaskar J. (2016). Evaluation of the flexural strength and microhardness of provisional crown and bridge materials fabricated by different methods. J. Indian. Prosthodont. Soc..

[9] Reeponmaha T., Angwaravong O., Angwarawong T. (2020). Comparison of fracture strength after thermo-mechanical aging between provisional crowns made with CAD/CAM and conventional method. J. Adv. Prosthodont..

[10] Schwantz J.K., Oliveira-Ogliari A., Meereis C.T., Leal F.B., Ogliari F.A., Moraes R.R. (2017). Characterization of Bis-Acryl Composite Resins for Provisional Restorations. Braz. Dent. J..

[11] Lee J., Clark S.R., Tantbirojn D., Korioth T.V.P., Hill A.E., Versluis A. (2022). Strength and stiffness of interim materials and interim fixed dental prostheses when tested at different loading rates. J. Prosthet. Dent..

[12] Coelho C., Calamote C., Pinto A.C., Esteves J.L., Ramos A., Escuin T., Souza J.C.M. (2021). Comparison of CAD-CAM and traditional chairside processing of 4-unit interim prostheses with and without cantilevers: Mechanics, fracture behavior, and finite element analysis. J. Prosthet. Dent..

[13] Taşın S., Ismatullaev A. (2022). Comparative evaluation of the effect of thermocycling on the mechanical properties of conventionally polymerized, CAD-CAM milled, and 3D-printed interim materials. J. Prosthet. Dent..

[14] Astudillo-Rubio D., Delgado-Gaete A., Bellot-Arcís C., Montiel-Company J.M., Pascual-Moscardó A., Almerich-Silla J.M. (2018). Mechanical properties of provisional dental materials: A systematic review and meta-analysis. PLoS ONE.

[15] Singh A., Garg S. (2016). Comparative Evaluation of Flexural Strength of Provisional Crown and Bridge Materials-An Invitro Study. J. Clin. Diagn. Res..

[16] Huettig F., Prutscher A., Goldammer C., Kreutzer C.A., Weber H. (2016). First clinical experiences with CAD/CAM-fabricated PMMA-based fixed dental prostheses as long-term temporaries. Clin. Oral. Investig..

[17] Alt V., Hannig M., Wöstmann B., Balkenhol M. (2011). Fracture strength of temporary fixed partial dentures: CAD/CAM versus directly fabricated restorations. Dent. Mater..

[18] Ellakany P., Aly N.M., Al-Harbi F. (2022). Accuracy of 3D Printed and Digital Casts Produced from Intraoral and Extraoral Scanners with Different Scanning Technologies: In Vitro Study. J. Prosthodont..

[19] Abad-Coronel C., Carrera E., Córdova N.M., Fajardo J.I., Aliaga P. (2021). Comparative Analysis of Fracture Resistance between CAD/CAM Materials for Interim Fixed Prosthesis. Materials.

[20] Alp G., Murat S., Yilmaz B. (2019). Comparison of Flexural Strength of Different CAD/CAM PMMA-Based Polymers. J. Prosthodont..

[21] Yao J., Li J., Wang Y., Huang H. (2014). Comparison of the flexural strength and marginal accuracy of traditional and CAD/CAM interim materials before and after thermal cycling. J. Prosthet. Dent..

[22] Dureja I., Yadav B., Malhotra P., Dabas N., Bhargava A., Pahwa R. (2018). A comparative evaluation of vertical marginal fit of provisional crowns fabricated by computer-aided design/computer-aided manufacturing technique and direct (intraoral technique) and flexural strength of the materials: An in vitro study. J. Indian. Prosthodont. Soc..

[23] Tahayeri A., Morgan M., Fugolin A.P., Bompolaki D., Athirasala A., Pfeifer C.S., Ferracane J.L., Bertassoni L.E. (2018). 3D printed versus conventionally cured provisional crown and bridge dental materials. Dent. Mater..

[24] Simoneti D.M., Pereira-Cenci T., dos Santos M.B.F. (2022). Comparison of material properties and biofilm formation in interim single crowns obtained by 3D printing and conventional methods. J. Prosthet. Dent..

[25] Henderson J.Y., Korioth T.V.P., Tantbirojn D., Versluis A. (2022). Failure load of milled, 3D-printed, and conventional chairside-dispensed interim 3-unit fixed dental prostheses. J. Prosthet. Dent..

[26] Ellakany P., Aly N.M., Al-Harbi F. (2022). A comparative study assessing the precision and trueness of digital and printed casts produced from several intraoral and extraoral scanners in full arch and short span (3-unit FPD) scanning: An in vitro study. J. Prosthodont..

[27] Lee W.S., Lee D.H., Lee K.B. (2017). Evaluation of internal fit of interim crown fabricated with CAD/CAM milling and 3D printing system. J. Adv. Prosthodont..

[28] Abduo J., Lyons K., Bennamoun M. (2014). Trends in computer-aided manufacturing in prosthodontics: A review of the available streams. Int. J. Dent..

[29] Darwish L.R., Al-Qady A., El-Wakad M.T., Farag M.M., Darwish R.R. (2022). Trends in 3D Printing Implants for Medical and Dental Applications. Ref. Modul. Mater. Sci. Mater. Eng..

[30] Aati S., Akram Z., Ngo H., Fawzy A.S. (2021). Development of 3D printed resin reinforced with modified ZrO_2_ nanoparticles for long-term provisional dental restorations. Dent. Mater..

[31] Ellakany P., Al-Harbi F., El Tantawi M., Mohsen C. (2022). Evaluation of the accuracy of digital and 3D-printed casts compared with conventional stone casts. J. Prosthet. Dent..

[32] Alharbi N., Osman R., Wismeijer D. (2016). Effects of build direction on the mechanical properties of 3D-printed complete coverage interim dental restorations. J. Prosthet. Dent..

[33] Alshamrani A.A., Raju R., Ellakwa A. (2022). Effect of Printing Layer Thickness and Postprinting Conditions on the Flexural Strength and Hardness of a 3D-Printed Resin. Biomed. Res. Int..

[34] Nakonieczny D.S., Kern F., Dufner L., Antonowicz M., Matus K. (2021). Alumina and Zirconia-Reinforced Polyamide PA-12 Composites for Biomedical Additive Manufacturing. Materials.

[35] Nakonieczny D.S., Kern F., Dufner L., Dubiel A., Antonowicz M., Matus K. (2021). Effect of calcination temperatures on the phase composition, morphology and thermal properties of ZrO_2_ and Al_2_O_3_ for biomedical applications modified with APTES (3-aminopropyltriethoxysilane). Materials.

[36] Kolb C., Gumpert K., Wolter H., Sextl G. (2020). Highly translucent dental resin composites through refractive index adaption using zirconium dioxide nanoparticles and organic functionalization. Dent. Mater..

[37] Aati S., Shrestha B., Fawzy A. (2022). Cytotoxicity and antimicrobial efficiency of ZrO_2_ nanoparticles reinforced 3D printed resins. Dent. Mater..

[38] Niem T., Youssef N., Wöstmann B. (2020). Influence of accelerated ageing on the physical properties of CAD/CAM restorative materials. Clin. Oral. Investig..

[39] Suralik K.M., Sun J., Chen C.-Y., Lee S.J. (2020). Effect of Fabrication Method on Fracture Strength of Provisional Implant-Supported Fixed Dental Prostheses. Prosthesis.

[40] Scotti C.K., Velo M.M.d.A.C., Rizzante F.A.P., Nascimento T.R.d.L., Mondelli R.F.L., Bombonatti J.F.S. (2020). Physical and surface properties of a 3D-printed composite resin for a digital workflow. J. Prosthet. Dent..

[41] Faul F., Erdfelder E., Lang A.G., Buchner A. (2007). G* Power 3: A flexible statistical power analysis program for the social, behavioral, and biomedical sciences. Behav. Res. Methods..

[42] Kadam P., Bhalerao S. (2010). Sample size calculation. Int. J. Ayurveda Res..

[43] Zimmermann M., Ender A., Attin T., Mehl A. (2020). Fracture load of three-unit full-contour fixed dental prostheses fabricated with subtractive and additive CAD/CAM technology. Clin. Oral Investig..

[44] Peñate L., Basilio J., Roig M., Mercadé M. (2015). Comparative study of interim materials for direct fixed dental prostheses and their fabrication with CAD/CAM technique. J. Prosthet. Dent..

[45] Wimmer T., Ender A., Roos M., Stawarczyk B. (2013). Fracture load of milled polymeric fixed dental prostheses as a function of connector cross-sectional areas. J. Prosthet. Dent..

[46] Karaokutan I., Sayin G., Kara O. (2015). In vitro study of fracture strength of provisional crown materials. J. Adv. Prosthodont..

[47] Park S.M., Park J.M., Kim S.K., Heo S.J., Koak J.Y. (2020). Flexural Strength of 3D-Printing Resin Materials for Provisional Fixed Dental Prostheses. Materials.

[48] Dentistry-Polymer-Based Crown and Veneering Materials.

[49] Pieniak D., Walczak A., Walczak M., Przystupa K., Niewczas A.M. (2020). Hardness and Wear Resistance of Dental Biomedical Nanomaterials in a Humid Environment with Non-Stationary Temperatures. Materials.

[50] Heintze S.D., Monreal D., Reinhardt M., Eser A., Peschke A., Reinshagen J., Rousson V. (2020). Fatigue resistance of all-ceramic fixed partial dentures—Fatigue tests and finite element analysis. Dent. Mater..

